# Supplementary material for: Clinical Utility of Prostate Health Index for Diagnosis of Prostate Cancer in Patients with PI-RADS 3 Lesions
Source: Cancers (Basel). 2022 Aug 29;14(17):4174. doi: 10.3390/cancers14174174 (PMC9454669; doi:10.3390/cancers14174174)
Supplement: Supplementary file 1 [file cancers-14-04174-s001.zip › cancers-1780149-supplementary.pdf]

cancers

MDPI

Supplementary Material: Clinical Utility of Prostate Health Index for Diagnosis of Prostate Cancer in Patients with PI-RADS 3 lesions

Chung Un Lee, Sang Min Lee, Jae Hoon Chung, Minyong Kang, Hyun Hwan Sung, Hwang Gyun Jeon, Byong Chang Jeong, Seong Il Seo, Seong Soo Jeon, Hyun Moo Lee and Wan Song *

Table S1. Baseline characteristics of patients with prostate cancer.

Variables	Overall PCa	Clinically		*p*
		Insignificant PCa	Significant PCa	
**No. of patients, *n* (%)**	**121 (100.0)**	**62 (51.2)**	**59 (48.8)**	
Age, years				0.625
Median (IQR)	65.0 (60.0–70.0)	65.0 (60.0-70.0)	64.0 (61.0-68.0)	
Mean (SD)	64.4 (7.3)	64.8 (7.3)	63.9 (7.1)	
Total PSA, ng/ml				0.389
Median (IQR)	6.85 (4.31–10.18)	6.74 (4.31-9.52)	6.98 (4.40-10.18)	
Mean (SD)	9.31 (9.21)	9.10 (8.39)	9.40 (10.1)	
Prostate volume, ml				0.850
Median (IQR)	34.6 (23.9–44.1)	34.6 (23.9-44.1)	34.5 (24.0-42.9)	
Mean (SD)	36.7 (18.0)	36.5 (15.2)	37.2 (18.6)	
PSAD				0.525
Median (IQR)	0.20 (0.10–0.23)	0.19 (0.10-0.21)	0.20 (0.11-0.23)	
Mean (SD)	0.25 (0.19)	0.25 (0.18)	0.25 (0.20)	
%fPSA				0.240
Median (IQR)	11.5 (9.9–18.3)	11.9 (10.2-18.3)	11.2 (9.9-17.8)	
Mean (SD)	14.1 (6.7)	14.5 (6.9)	13.3 (7.4)	
p2PSA, pg/ml				0.287
Median (IQR)	24.24 (18.62–38.74)	22.60 (18.62-37.30)	26.41 (21.33-38.74)	
Mean (SD)	27.62 (7.49)	25.30 (8.77)	29.14 (7.67)	
%p2PSA				0.182
Median (IQR)	1.81 (1.49–2.23)	1.74 (1.49-2.06)	1.91 (1.64-2.23)	
Mean (SD)	2.05 (1.55)	2.01 (1.55)	2.17 (1.78)	
PHI				0.224
Median (IQR)	46.5 (35.0–62.1)	44.6 (35.0-57.8)	48.1 (38.1-62.1)	
Mean (SD)	51.6 (19.5)	49.3 (18.1)	52.9 (20.1)	
Biopsy Gleason grade, *n* (%)				
6	62 (51.2)	62 (100.0)		
7 (3+4)	47 (38.9)		47 (79.7)	
8	12 (9.9)		12 (20.3)	

Table S2. Cancer detection on target, off target and whole prostate.

Variables	N = 392
Systemic biopsy	
Benign	337 (86.0)
Gleason grade, *n* (%)	
6	35 (8.9)
7 (3+4)	18 (4.6)
8	2 (0.5)
Target biopsy	
Benign	271 (69.1)
Gleason grade, *n* (%)	

6	62 (15.8)
7 (3+4)	47 (12.0)
8	12 (3.1)
Systemic + target biopsy	
Benign	253 (64.5)
Gleason grade, *n* (%)	
6	72 (18.4)
7 (3+4)	55 (14.0)
8	12 (3.1)
